# Clinical presentations of Hepatitis E: A clinical review with representative case histories

**DOI:** 10.1016/j.clinre.2019.01.005

**Published:** 2019-11

**Authors:** Abhishek Chauhan, Gwilym Webb, James Ferguson

**Affiliations:** a NIHR Birmingham Biomedical Research Centre, United Kingdom; b Liver unit, University Hospitals Birmingham, United Kingdom; c Institute of Immunology and Immunotherapy, University of Birmingham, United Kingdom

**Keywords:** Hepatitis E, Acute-on-chronic liver failure, liver transplantation, acute decompensation, viral hepatitis

## Abstract

Hepatitis E virus (HEV) typically causes an acute, self-limiting hepatitis and is among the commonest cause of such presentations. Hepatitis E viral infection is also increasingly recognized as a cause of chronic hepatitis amongst the immunocompromised, particularly amongst solid organ transplant recipients. Chronic HEV infection remains an underdiagnosed disease and chronic infection can lead to rapidly progressive liver fibrosis and cirrhosis. This review examines current understanding of the HEV. We illustrate typical clinical presentations, management strategies [(based upon guidelines from both the British Transplant Society (BTS) and European Association for the study of liver (EASL)] and outcomes of HEV infection in different cohorts of patients by highlighting select transplant and non-transplant patient cases, from one of the largest tertiary Hepatology centres in Europe.

## Introduction

### What is already known?

Hepatitis E virus (HEV) infection which was traditionally thought to be a condition mainly affecting travellers and populations within developing nations is now being increasingly detected amongst Western cohorts of patients. An increase in both incidence and prevalence in the last decade has been noted and immunosuppressed individuals such as solid organ transplant recipients are at significant risk of developing chronic hepatitis and liver fibrosis.

### What this review adds?

We build upon our current understanding about the pathobiology and epidemiology of this virus by appraising and presenting the latest evidence surrounding HEV infection. We next illustrate clinical presentation, management strategies and outcomes of HEV infection in different cohorts of patients using select patient cases from a large European tertiary Hepatology centre.

### Who will this benefit from this review?

This will be of value to all gastroenterologists and hepatologists involved in the care of patients presenting with acute hepatitis, as well as those caring for the chronically immunosuppressed including transplant recipients. We highlight HEV infection as an increasingly relevant differential diagnosis in immunosuppressed individuals with deranged liver function tests and even in those with suspected drug induced liver injury.

## Hepatitis E virology

### The virus

Hepatitis E virus (HEV) infection is estimated to be the commonest cause of acute hepatitis in the world [1]. Khuroo et al first identified HEV as an unknown non-A, non-B virus during an outbreak of jaundice in the winter of 1978–1979 in Kashmir [2]. Subsequently a Russian army doctor provided the first evidence of faeco-oral transmission of this virus. During the Soviet occupation of Afghanistan in the 1980s to investigate the nature of an outbreak of unexplained, non-A, non-B acute hepatitis at a military encampment, he ingested pooled faecal extract from affected soldiers, and subsequently developed the illness. His serum contained particles that reacted with saved sera from those previously diagnosed with the new hepatitis, thus confirming faeco-oral transmission of the virus [3]. Others have subsequently emulated the procedure [4].

HEV infection is in the majority of patients a self-limiting infection causing a hepatitis, which may be accompanied by jaundice. Typical symptoms include anorexia, malaise, nausea and jaundice; in the immunocompetent individual, the infection and symptoms normally resolve spontaneously within 4–6 weeks and the median incubation period between infection and development of symptoms is 40 days [5]. Although the majority of patients who become infected with HEV remain asymptomatic [1], HEV infection in certain groups of patients may cause more fulminant disease or progressive fibrosis. Those with pre-existent chronic liver disease for instance can develop acute on chronic liver failure; infection during the third trimester of pregnancy is associated with high maternal and foetal mortality [6]. HEV is now increasingly identified as a cause of chronic infection in immunocompromised patients including transplant patients [7]. The frequent and rapid rate of progression to hepatic fibrosis and cirrhosis in solid organ transplant recipients (with 60% of those infected developing chronic hepatitis [8] and 10% of all HEV infected transplant recipients progressing to cirrhosis [9]), who are often on a complex regime of immuno-suppression, makes timely recognition and prompt treatment particularly important [10].

There are 7 genotypes (G1–7) of this hepatotropic RNA virus [11]; G1 to G4 and G7 are capable of human infection [12]; genotypes 5 and 6 only infect animals [13]. G1 and G2 are exclusively human pathogens and responsible for waterborne outbreaks in developing countries [5]; G3 and G4 are enzootic in swine [14].

The genus *Sus,* which includes pigs reared for human consumption as well as other species including wild boar, provides a large reservoir for G3 and G4 [14]. HEV causes no morbidity in pigs, and although effective porcine vaccines exist, grounds for their use in pigs (to effectively reduce the reservoir for human infection) against HEV are thus weak [15]. This remains an important consideration as foodborne zoonosis represents the commonest mode of HEV infection (G3 and G4) in the Western world [15]. Infected porcine meat infects humans as end line hosts but human-to-human transmission of G3 and G4 appears restricted to blood transfusion and organ transplantation [14].

## Epidemiology and transmission

HEV infection is a significant public health problem: the World Health Organisation estimates that there are about 20 million HEV infections worldwide per year [5] with the majority of the disease burden being secondary to HEV G1/G2 infection [20] HEV infection causes a clinically identifiable acute liver injury in 3.5 million and approximately 56,000 deaths (2800 per 1,000,000 infections) [21], [22].

**Figure 1:**
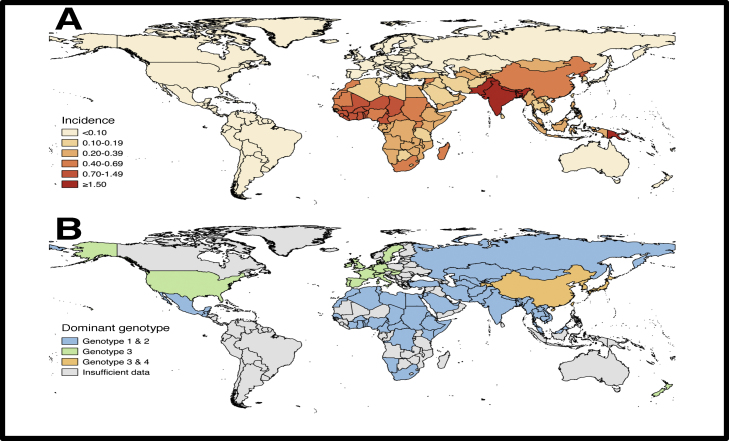
A. Age-standardised disability-adjusted life-year rates (per 100,000 per year) attributable to hepatitis E virus (2013, by country). Adapted from data provided in Stanaway et al. [22]. B Dominant genotypes of clinical cases of hepatits E infection. Adapted from Kamar et al [1].

G1 and G2 HEV are endemic in certain developing countries and associated with water-borne outbreaks. G1 is found in Asia and Africa; G2 is less common and found in Mexico and Africa [7] (Fig. 1). The majority of HEV infections in the developing world are thus due to HEV G1 or G2 but the true burden of disease is not known [5], [7]. The genotypes responsible for tropical or endemic HEV (G1 and G2) mainly affect younger people compared to the HEV G3 infections which are predominantly found in middle aged men [18]. Outbreaks in the West remain relatively rare but have been reported in instances of common-source foodborne outbreaks [23].

HEV G3 and G4 are the zoonotic HEV genotypes [15]; G4 is mainly found in Asia [7]. HEV G3 remains the dominant genotype responsible for autochthonous (locally acquired) transmission in the West [5]. There is marked variability amongst reported Anti-HEV G3 seroprevalence in mainland Europe, ranging from 0.6% to 52% [24] and between 3–16% in the UK [5]. Considerable geographical variability is observed in HEV G3 infection within countries, for example in France, there is a much higher reported seroprevalence in the southwest, southeast and northeast of the country [5], [25]. Contaminated food stuffs are thought responsible for this regional variation: The mechanism of transmission of HEV G3 and G4 is predominantly food or blood products [1].

Although de novo cases of HEV are rarely reported in the United States (US), a 2009 study placed the seroprevalence of anti-HEV IgG in the US population at 21% [26]; meat consumption was a significant risk factor for seropositivity. Guidelines published by the British Transplant Society (BTS) estimate that 1 in 2500 blood donations are HEV RNA positive and the UK Advisory Committee for the Safety of Blood, Tissues and Organs thus recommend universal screening for all blood components for HEV (with particular care taken not to transfuse some immunosuppressed groups with HEV + bloods) [27]. It is important to note that HEV infected donor blood is on the whole rare and that even infected blood tends to contain low levels of virus; this is in most cases insufficient to cause recipient infection [14]. Tedder et al demonstrated that the lowest viral dose that resulted in infection was 2 × 10^4^ IU and that 55% of all blood components containing this dose or more transmitted infection [14] but go on to suggest that for the vast majority of solid organ transplant recipients, dietary risks far exceed the risks from transfusion from unscreened donors [14], a large Canadian study further confirmed that the risk of acquiring HEV through an infected blood donation is low [28]. The biggest risk factors for acquiring HEV in the West are thus being on haemodialysis [29], consumption of infected meat [30], [31] or for workers who come into contact with swine regularly [32], [33]. Direct transmission from consumption of infected wild boar [17], pig [34] and deer [35] meat has been clearly demonstrated and both the BTS and EASL recommend advising all solid organ transplant recipients regarding the risk from undercooked meat particularly pork [36], [37].

## Diagnostic testing

**Figure 2:**
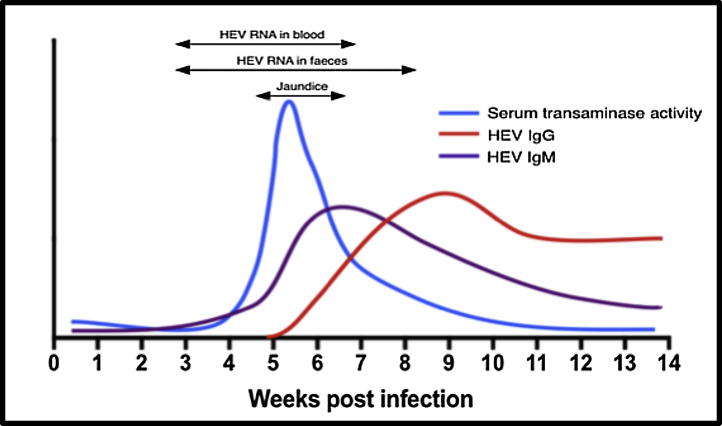
Graph demonstrating how HEV viral load varies with respect to development of jaundice and seroconversion (adapted from Dalton et al. [38]).

HEV can be detected by either directly demonstrating the viral genome in biological samples such as in stool or blood, or indirectly by testing for HEV-specific IgM or IgG antibodies [38]. Anti-HEV IgM seropositivity precedes the development of IgG, peaking at the same time as peak ALT and lasting up to 5 months after the initial disease onset; IgG antibodies on the other hand appear after IgM but can last beyond a year, occasionally disappearing before the one year mark [38] (Fig. 2).

Re-infection with HEV will result in repeated seroconversions which makes determining duration of infection difficult, thus estimating chronicity of infection from serology alone is challenging; this is particularly an issue in countries where the virus is endemic [1]. The predictive accuracy of positive IgM anti-HEV antibody assays in diagnosing HEV in low endemicity areas and in patients with atypical presentations is poor [39]. Additionally, there exists a wide variability in diagnostic accuracy between the different commercially available immunoassays [40]. A study from Taiwan examining both IgG and IgM anti-HEV revealed that whilst most assays performed excellently with regards to negative predictive value (98.4%-100%), this was not however the case with positive predictive value as a wide intra-test variability in specificity (62.9–95.6%) and sensitivity (66.7–93.3%) was seen [41]. Furthermore, false positives for HEV serology have been reported with other forms of acute viral hepatitis [42] and even systemic EBV infection [43]. For these reasons, the gold standard for confirming HEV remains detecting the virus in biological samples via polymerase chain reaction (PCR). Broadly speaking, the virus is detectable in stool a week before symptoms begin and remains detectable for up to 6 weeks, the window for detection in the serum is smaller [38]. An undetectable viral RNA load does not necessarily rule out recent HEV infection as timely sample collection, early patient presentation and local epidemiology influences the sensitivity of available molecular tests [1]. Direct viral detection is of particular importance in patients on immunosuppressive medications as the seroconversion process in such a cohort is often impaired and thus relying on antibodies alone for diagnosis may lead to a false negative results [44].

## Clinical presentations

### Acute hepatitis in the immunocompetent host

Acute HEV infection (regardless of causative genotype) is silent and self-limiting in the vast majority; Kamar at al estimate that both in sporadic infection and outbreaks asymptomatic individuals with HEV infection outnumber the symptomatic by almost 4 to 1 [1]. When it does manifest clinically, acute HEV infection presents in an identical manner to other viral hepatitides including a short non-specific prodromal phase characterised by influenza-like symptoms, myalgia and malaise followed by jaundice, itching and dark urine [18]. Peak serum alanine transaminase (ALT) activity occurs around 6 weeks after infection; the incubation period for symptomatic acute HEV infection is between three and eight weeks [16]. Tropical and endemic variants of the disease (G1 and G2) are associated with a more aggressive biochemical [18] and clinical phenotype with 16% of those contracting the virus exhibiting signs of an acute inflammatory hepatitis [45] compared to only 2% in those infected with G3 or G4 [46]. Progression of acute HEV infection to fulminant liver failure remains rare and there are only two recorded examples of HEV induced acute fulminant failure requiring emergency transplantation [47], [48]. Given the potential similarity in presentation, some authors recommend testing all suspected cases of drug induced liver injury for HEV infection [49]. Finally, it is worth noting that in one centre's experience the triad of bilateral shoulder pain in a middle-aged male with abnormal LFTs is highly predictive of HEV infection [50].

### Treatment

The majority of acute HEV infections are self-limiting and thus do not require treatment [5], [18]. Ribavirin has been used to treat cases of severe acute and ACLF due to HEV infection with varying degrees of success in areas where G1/G2 infection are endemic [51]. Treatment of acute HEV G3 infection using ribavirin has also been described [52]; more recently however cases of ribavirin resistance and treatment failure have been noted, these seem to relate to either ribavirin dose reduction due to side effects such as anaemia or *HEV* mutations (*G1634R* mutation in the HEV ORF1 protein) [5]. In all cases, clear thresholds for the initiation of ribavirin therapy remain undefined. Table 1

**Table 1:** Summary of known relative virulence, species affected and mode of transmission for different strains of HEV [5], [15], [16], [17], [18], [19].

Genotype	Virulence	Species affected	Dominant mode of transmission
G1	+++	Humans	Faeco-oral
G2	+++	Humans	Faeco-oral
G3	+	Humans and swine	Zoonotic transmission from consumption of infected meat and via blood transfusion from affected donor
G4	++	Humans and swine	
G5	unknown	Swine	No reported human cases One reported case of transmission to humans from infected camel meat/milk
G6	unknown	Swine	
G7	unknown	Camels	

### Acute hepatitis in the immunocompromised host

Acute HEV in immunocompromised patients generally presents asymptomatically [53], if symptoms do occur these are non-specific and include jaundice, fatigue, diarrhoea and myalgia. Predictably, the necroinflammatory T-cell mediated immune response, which accompanies HEV infection, is more aggressive in the immunocompetent individual. Thus whilst serum transaminase activity in non-immunosuppressed individuals at presentation is often greater than 1000 IU/L; it is often in the 100–300 IU/L range in the immunosuppressed [53]. The natural history of acute infection in solid organ transplant recipients suggests that specific treatment or immunosuppressant modification is not always necessary, because a significant number will clear the virus spontaneously [37].

## Treatment

Initial management of acute HEV infection is suggested to be careful monitoring of HEV RNA levels, serology and hepatic enzyme activity. The BTS and EASL both advocate an approach of careful reduction in immunosuppressive medication where feasible [36], [37], except when HEV infection is associated with extrahepatic manifestations where the BTS guidelines recommend early treatment with ribavirin. It is noteworthy however that a study from Holland found that immunosuppression reduction exhibited greater efficacy in HEV eradication than ribavirin in patients with extrahepatic HEV manifestations [54].

### Acute hepatitis in pregnancy

A group of patients that merit specific mention in the context of acute HEV G1 infection is pregnant women. Infection particularly in the third trimester is associated with devastating maternal and foetal outcomes [5]. A study from India confirmed that outcomes for pregnant patients with acute viral hepatitis were far poorer both for the mother and foetus when the infecting virus was HEV compared to hepatitis B or C [6], although the aetiopathogenesis behind this is unclear; the relative immunocompromised state that pregnancy confers is likely culpable [5]. Treatment is primarily supportive, however a recombinant protein based vaccine has been found to be safe and effective in phase 2 and phase 3 trials in China [55].

### Chronic hepatitis in the immunocompetent

HEV chronicity as defined by viral persistence for at least 6 months [5] is primarily noted in patients with either acquired or inherited immunodeficiency. Although isolated reports of immunocompetent individuals developing chronic HEV infection have been described [56], [57] an antecedent sometimes occult history of an immunosuppressed state is often noted [57].

## Chronic hepatitis in the immunocompromised host

**Figure 3:**
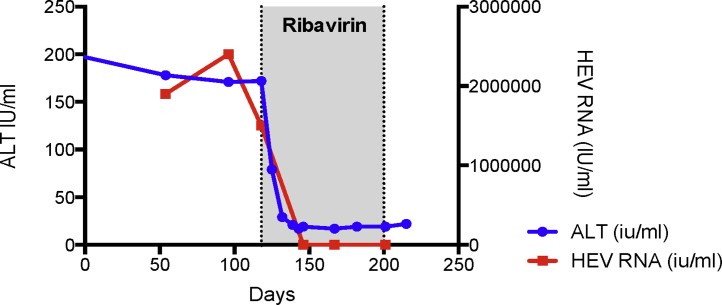
HEV infection drives the rapid development of cirrhosis in patients on immunosuppressive medication. Patient who had undergone simultaneous heart and kidney transplantation in 2002 and on triple immunosuppression (mycophenolate mofetil, tacrolimus and prednisolone) was referred to liver medicine after having been noted to have deranged liver function. The first noted elevations in serum alanine aminotransferase (ALT) activity was recorded as 94IU/l which coincided with symptoms of malaise, lethargy and diarrhoeal symptoms. The referring team screened him for liver disease, stopped his statin medication and presumptively treated him for cytomegalovirus hepatitis. When his liver biochemistry failed to improve he was referred and seen in liver clinic. He had established liver cirrhosis with significant portal hypertension with median transient elastography at his index liver appointment > 20 kPa. He was subsequently found to have a HEV load of over 2.4 × 106 IU/mL with compatible serology (HEV IgM and IgG positive). Ribavirin was initiated which resulted in a virological and serum ALT response. He is now being followed up as a well-compensated cirrhotic patient by liver outpatient services. The time course from the first biochemical abnormality being detected, to the likely development of chronicity through to confirmed cirrhosis was only 16 months.

Given the rapidity with which immunosuppressed patients who contract chronic HEV may develop end-stage liver disease it is imperative to consider chronic HEV infection in those with evidence of liver injury (Fig. 3). Once infected between 46 and 80% of solid organ transplant recipients are ultimately unable to spontaneously clear the virus and are thus at risk of chronicity [10], [44], [58]; significant hepatic fibrosis can develop as early as 12 months after HEV infection [44]. Immunosuppression, iatrogenic or otherwise, results in HEV persistence, which then drives iterative bouts of hepatic necro-inflammation, characterized clinically by chronically raised ALT activity and eventually the development of hepatic fibrosis and cirrhosis. Risk factors for the development of chronicity in solid organ transplant recipients include the use of tacrolimus and the transplanted organ being a liver [53].

Chronic HEV infection may occur in any immunocompromised patient including HIV positive patients [53], patients with chronic granulomatous conditions or connective tissue disorders like SLE [59] and as illustrated above, immunosuppressed organ transplant recipients [53]. The incubation period for the virus in the context of immunosuppression is longer than seen in immunocompetent hosts at 60 days, with chronicity itself being defined by viral persistence after the acute phase for either 3 or 6 months [5]. Chronic HEV infection in immunocompromised patients is almost exclusively secondary to HEV G3 infection; one case of chronic HEV G4 infection has been noted but none due to HEV G1 or G2 [5].

### Treatment of solid organ transplant recipients

Management of HEV infection in immunosuppressed solid organ transplant recipients represents a unique challenge. Commonly used immunosuppressive medications for solid organ transplant recipients have varying effects on HEV replication, calcineurin inhibitors such as tacrolimus and ciclosporin for instance have been shown to enhance HEV replication in vitro whilst mycophenolate mofetil seems to inhibit HEV replication [60]. Mechanistic target of rapamycin (mTOR) inhibitors may perpetuate HEV infection [61]. Interestingly steroids have actually been noted to counteract the powerful hepatic necroinflammatory response that HEV infection can occasionally elicit, whilst simultaneously allowing for viral clearance [62], although their therapeutic potential role is unclear (Fig. 3).

**Figure 4:**
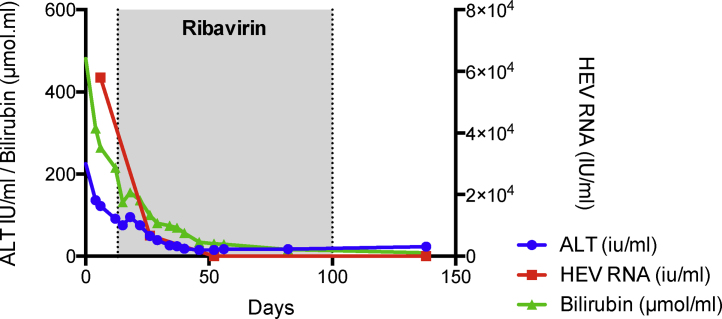
HEV infection drives the development of acute on chronic liver failure in patients with established cirrhosis. A 54-year-old male who was active on the liver transplant waiting list for non-alcoholic steatohepatitis associated cirrhosis presented with a 10-day history of acute decompensation manifested by the development of jaundice and worsening ascites. His complicated medical history was significant for small bowel Crohn's disease; and he had recently been commenced on an anti-TNFa monoclonal antibody (adalimumab; Humira®) and prednisolone. Testing confirmed acute HEV G3 infection with HEV detectable in both blood and stool by PCR. In contrast to the non-cirrhotic transplant recipient mentioned above, acute HEV infection in this instance resulted in significant liver dysfunction exemplified by coagulopathy, jaundice and ascites. The patient recompensated with ribavirin and exhibited a SVR. Stool HEV PCR was negative at 3 and 6 months post treatment.

In our centre, we have had 36 cases of serologically confirmed chronic HEV in solid organ transplant recipients. Initial management for most of our cases was a reduction in immunosuppression. Over half (55%–20 patients) cleared HEV infection by this measure, the remainder had a significant residual viraemia and were treated with ribavirin (16 patients). A quarter of solid organ transplant recipients treated at our centre for chronic HEV infection exhibited treatment failure and thus required at least one further course of ribavirin; in all of these patients HEV relapse was noted due to persistent HEV stool shedding at three months and all related to a ribavirin dose reduction (due to ribavirin side effects) (Fig. 4).

Consistent with our practice, the BTS and EASL guidelines [36], [37] suggest that if feasible the initial treatment for HEV infection in solid organ transplant recipients should be a reduction in the dose of immunosuppression; this measure clears HEV infection in about a third of infected patients [8]. Patients that present with severe liver dysfunction (coagulopathy, jaundice or encephalopathy) or exhibit viral persistence as evidenced by infection lasting greater than three months despite a reduction in immunosuppression need further treatment [36]. The BTS recommend ribavirin as the first line agent [36], [63]. The dose needs to adjusted according to renal function and haemoglobin levels (EASL guidelines), dose dependent anaemia remains a relative contraindication and was a common reason for therapy cessation at our centre. Once started, treatment normally lasts between 3 to 6 months; negative stool samples and serum viral PCR will help dictate when to stop treatment as continued shedding of HEV in stool is an important factor in predicting relapse after ribavirin treatment. An early response to ribavirin treatment helps predict long-term clearance and can be used to guide length of treatment [64]. Kamar et al found that treatment with ribavirin in solid organ transplant recipients at a median dose of 600 mg/day for three months resulted in an SVR of 78%, the majority of relapsers attained SVR after 6 months of ribavirin monotherapy [37], [63]. The EASL guidelines thus suggest that relapse after 3 months of ribavirin therapy should be treated with a longer 6-month course of ribavirin. In cases of treatment failure, as gauged by viral persistence in the stools or serum after completion of ribavirin therapy, EASL suggest a trial of pegylated interferon for 3 months in liver transplant recipients [37]; pegylated interferon is however contraindicated in heart, lung, kidney and pancreas recipients due to the relatively greater risk of ribavirin triggered acute rejection [65].

### Acute on chronic liver failure

Acute HEV infection in patients with pre-existing chronic liver disease can result in acute-on-chronic liver failure (ACLF) [66] (Fig. 4). The infecting HEV genotype appears to dictate severity or degree of liver injury encountered as although ACLF has been described with HEV G3 in a European cohort of patients, no difference in patient outcome in terms of mortality compared to other causes of ACLF [67] was noted. Contrastingly studies from China and the Indian subcontinent where the responsible genotype was always either HEV G1 or G2 [66] reveal a far worse outcome. Composite mortality was reported as up to 67% with a median of 34% [66]. Shrestha et al in fact report that over a fifth of all cases of ACLF in Asian countries are due to acute HEV infection [51].

## Treatment

Acute HEV infection in patients with established cirrhosis can result in the development of ACLF. The BTS suggest the early use of ribavirin in patients with end stage liver disease awaiting liver transplantation [36]. In the case discussed (Fig. 4) ribavirin cleared HEV infection sufficiently to allow hepatic recompensation.

### Extrahepatic manifestations

**Figure 5:**
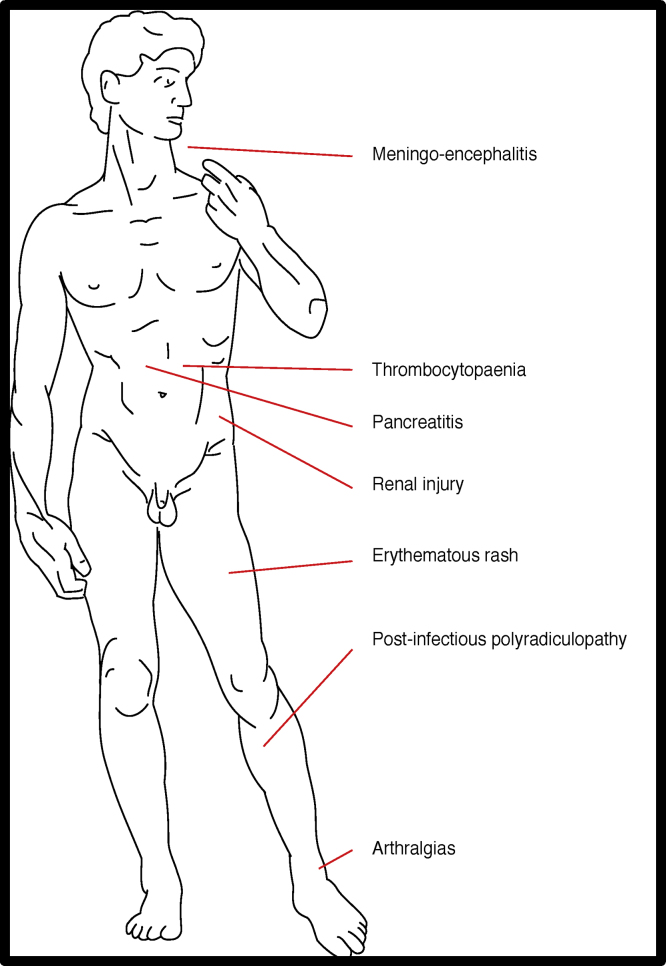
Summary of extrahepatic manifestations of HEV infection.

Although HEV has been associated with a variety of extrahepatic syndromes, a causal link for most of these is yet to be established [68] (Fig. 5). Acute pancreatitis in patients with fulminant viral hepatitis has been previously been described, but this is mainly in patients with hepatitis A, B or C. More recently, HEV has been noted to cause acute pancreatitis [69]. Most reports of HEV associated pancreatitis come from South Asia due to G1; no reports of G3 resulting in pancreatitis have been described [68].

Over 100 cases describing the neurological sequelae of HEV infection have also been reported [70]. The neurological injury that accompanies HEV infection manifests with a variety of symptomology ranging from post-infectious polyradiculoneuropathy (Guillain–Barré syndrome; GBS) to neuralgic amyotrophy and meningo-encephalitis [70]. It seems likely that the neurological insult in GBS and neuralgic amyotrophy is postinfectious and immune mediated; this is in line with what is currently known about the pathophysiology of these conditions [68], [70]. HEV meningo-encephalitis is in contrast thought to be a consequence of the direct neurotoxic effect of the virus [70], [71]. Definitive causality is yet to be established in either of these neurological conditions but the presence of HEV RNA in the CSF of patients with meningo-encephalitis arguably confirms the neurotropic nature of the virus [68], [72], [73].

Both acute and chronic HEV infection are also associated with kidney injury [68]. The causative renal pathology is variable as kidney biopsies of patients who were HEV infected and presented with renal abnormalities reveal histological features of membranous glomerulonephritis, membranoproliferative glomerulonephritis and even relapses of IgA nephropathy [68]. Aside from one report of HEV G1 causing renal dysfunction the majority of reported cases of HEV driven renal dysfunction are due to HEV G3 [71]. Again definitive causality is yet to be established but HEV clearance was associated with normalization of renal function [68]. A possible mechanism via which renal dysfunction occurs in HEV infected patients is through the development of cryoglobulinaemia [68], [71].

## Treatment

Comprehensive data accurately defining management of extrahepatic HEV are currently lacking [68]. Anecdotal evidence suggests early treatment with ribavirin may alter the natural history of the disease [74] and thus the BTS suggest early treatment with ribavirin be initiated.

## Conclusion

Infection with HEV remains a global cause of mortality and morbidity. The last 10 years have resulted in a far greater understanding about the pathobiology of this virus; particularly relevant to the western populace and indeed the wider transplanted community is the propensity for HEV infection to drive rapid liver fibrosis and thus end stage liver disease in the immunosuppressed. Prompt recognition and early intervention in such groups of patients remains key to effective management. An important question to address going forward is whether vaccination may be beneficial in patients where HEV infection is associated with particularly bad outcomes such as the immunosuppressed or patients with chronic liver disease, although vaccines for HEV are in trials; the efficacy of vaccination in such groups of patients is yet to be established [37]. Given the fact that the risk of contracting the virus is greater from infected foodstuff than even infected blood transfusion [14], dietary measures including avoidance of undercooked meat and shellfish in at risk populations such as immunosuppressed transplant recipients would likely be beneficial in reducing hepatitis E transmission [37].

## Funding

Although no specific funding was received for this project, AC has benefitted from a Wellcome trust clinical research training fellowship and GJW has benefitted from UK Medical Research Council Clinical Research Fellowship. This paper presents independent research supported by the NIHR Birmingham Biomedical Research Centre at the University Hospitals Birmingham NHS Foundation Trust and the University of Birmingham. The views expressed are those of the author(s) and not necessarily those of the NHS, the NIHR or the Department of Health.

## Contributions

JF originally conceived the article which was then modified in response to suggestions from each of the other authors. The manuscript was written by AC. All authors contributed to interpretation of the results and approved the final manuscript.

## NIHR Disclaimer

This report presents independent research funded by the National Institute for Health Research (NIHR). The views expressed are those of the authors(s) and not necessarily those of the NHS, the NIHR or the Department of Health

## Disclosure of interest

The authors declare that they have no competing interest.
